# Preoperative disseminated intravascular coagulation complicated by thoracic aortic aneurysm treated using recombinant human soluble thrombomodulin: A case report

**DOI:** 10.1097/MD.0000000000025044

**Published:** 2021-03-05

**Authors:** Yoshinori Tanigawa, Yasutaka Yamada, Kimihide Nakamura, Tomoko Yamashita, Akira Nakagawachi, Yoshiro Sakaguchi

**Affiliations:** a Department of Anesthesiology; b Intensive Care Unit, Saga Medical School Hospital, Faculty of Medicine, Saga University, Saga, Japan.

**Keywords:** case report, disseminated intravascular coagulation, recombinant human soluble thrombomodulin, thoracic aortic aneurysm

## Abstract

**Rationale:**

Chronic disseminated intravascular coagulation (DIC) associated with thoracic aortic aneurysm is characterized by enhanced fibrinolysis and is thought to be stable in the compensated/asymptomatic stage, with few bleeding symptoms. However, DIC can lead to decompensated/hemorrhagic stage disseminated intravascular coagulation, resulting in severe bleeding diathesis, and there is currently no established strategy for treatment of DIC in aortic aneurysms.

**Patient concerns:**

A 77-year-old woman underwent angiography and cardiac catheterization, before descending aortic replacement surgery. She developed DIC in postprocedure week 2 with extensive, uncontrollable massive subcutaneous hemorrhage.

**Diagnoses:**

Her acute-phase DIC score was 7 points, and the risk of mortality within 30 days after surgery according to the JapanSCORE was estimated to be 33.6%.

**Interventions:**

Therapy was a combination of recombinant human soluble thrombomodulin (rhTM) and an aortic stent-graft treatment.

**Outcomes:**

Short-term improvements were seen in both DIC and bleeding diathesis. The thoracic aortic aneurysm with severe DIC was eventually corrected by administration of rhTM.

**Lessons:**

We report the use of rhTM as an effective, novel anticoagulant drug with anti-inflammatory activity for treating DIC with suppressed fibrinolysis, which is typically associated with sepsis. In patients with a high hemorrhagic diathesis, in whom preoperative control of DIC cannot be achieved with conventional anticoagulation and radical surgical repair cannot be performed, a combination of rhTM and endovascular therapy may be a powerful new treatment option.

## 1 Introduction

Disseminated intravascular coagulation (DIC) syndrome associated with thoracic aortic aneurysm is a complication observed in 5.7% of all aortic aneurysm cases; however, it is reportedly stable in the decompensated/asymptomatic or chronic stage, which exhibits few bleeding symptoms.^[1,2]^ The clinical manifestations of aortic aneurysm-induced DIC are marked fibrinolytic activation that is more than commensurate with coagulation activation and, in rare cases, leads to DIC during the necrotic/hemorrhagic phase, which can lead to hemostatic difficulties and potentially fatal bleeding.^[3–6]^ Although the fundamental treatment of aortic aneurysms is surgical resection, the risk of bleeding is extremely high in cases wherein DIC is a complication. Therefore, preoperative control of DIC is important.

Unfortunately, there is no established strategy for treatment of DIC in aortic aneurysms. Different drugs have been used in the past to treat DIC; however, it has been shown that recombinant human soluble thrombomodulin (rhTM) can be safely used for all types of DIC.

rhTM is a drug formulation created by genetic transformation of the active extracellular domain of thrombomodulin, a glycoprotein present on the endothelial cell surface. It has 3 activities: anticoagulation activity by combining with thrombin produced in excess in the blood vessel to form a thrombin–TM complex, thereby antagonizing the effects of thrombin (such as fibrin-forming and platelet-activating abilities); anti-inflammatory activity; and vascular endothelial protection activity, derived from the inhibitory effect against the production of plasminogen activator inhibitor-1 (PAI-1) released from the injured vascular wall.^[7–9]^ The thrombin–TM complex is also reported to be effective in the treatment of chronic DIC because it inhibits excessive fibrinolysis by activating thrombin-activated fibrinolysis inhibitors.^[7,8,10]^

Here, we report a case with a thoracic aortic aneurysm in which preoperative administration of rhTM markedly improved DIC in a patient with the preoperative complication of hyperfibrinolytic DIC and marked bleeding symptoms.

## 2 Case presentation

Written, informed consent has been obtained in full, from the patient and her family, for publication of this case report.

### 2.1 Current medical history

**Figure 1:**
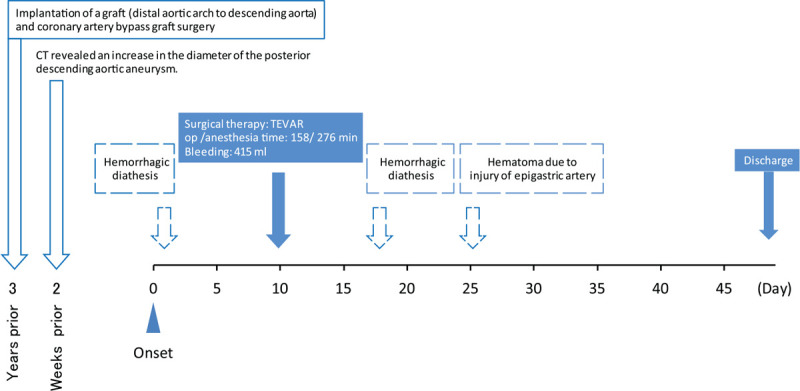
Historical information and clinical course of treatments. TEVAR = thoracic endovascular aortic repair.

The patient was a 77-year-old woman, 145 cm tall and weighing 35 kg. Implantation of a graft (distal aortic arch to descending aorta) and coronary artery bypass graft surgery (left internal mammary artery to left anterior descending coronary artery) were performed for impending rupture of an aortic aneurysm 3 years prior. Follow-up computed tomography revealed an increase in the diameter of the posterior descending aortic aneurysm, and repeat surgery was scheduled. Upon preoperatively performing angiography and cardiac catheterization, hemostasis was difficult to achieve at the puncture sites; extensive subcutaneous hemorrhage was identified. We empirically suspected that these symptoms were due to aortic aneurysm-related DIC, malignancy, hematologic tumor, or collagen disease; therefore, we decided to admit the patient to the hospital for a closer examination (Fig. 1).

### 2.2 Findings on admission

The patient was lucid with a blood pressure of 132/80 mm Hg and a normal pulse rate of 74 BPM. A grade II systolic murmur, based on the Levine grading system, was detected at the left sternal border in the fourth intercostal space. Spots of subcutaneous hemorrhage were observed at the extremities, other than at the puncture sites.

### 2.3 Laboratory findings on admission

The test results were as follows: white blood cell count, 3500/μL; hemoglobin and platelet level 10.5 and 105,000/μL, respectively; prothrombin time-international normalized ratio, 0.98; activated partial thromboplastin time, 42.3 seconds; fibrinogen level, 113 mg/dL; fibrinogen degradation product (FDP) level, 76 μg/mL; D-dimer level, 28 μg/mL, plasmin-α2-plasmin inhibitor complex (PIC) level, 7.2 μg/mL, thrombin–antithrombin complex (TAT) level, 49 ng/mL, aspartate aminotransferase level, 20 IU/L, alanine transaminase level, 11 IU/L, lactate dehydrogenase level, 222 IU/L, blood urea nitrogen level, 20 mg/dL, creatinine level, 1.2 mg/dL, and estimated glomerular filtration rate, 35 mL/min. Significant increases in TAT and PIC, as well as increases in FDP and D-dimer, were observed; this indicated remarkable fibrinolytic activation. Based on the diagnostic criteria for DIC by the Japanese Association of Acute Medicine, the score on admission was 7 points (normal range: 0–1).^[11]^

### 2.4 Computed tomography results

From the aortic arch to the descending aortic junction, as well as from the renal to the common iliac artery branch, the aorta showed nodular-like expansion. The descending aorta exhibited a maximum diameter of 55 mm, with a tendency toward expansion.

### 2.5 Clinical course after admission

**Figure 2:**
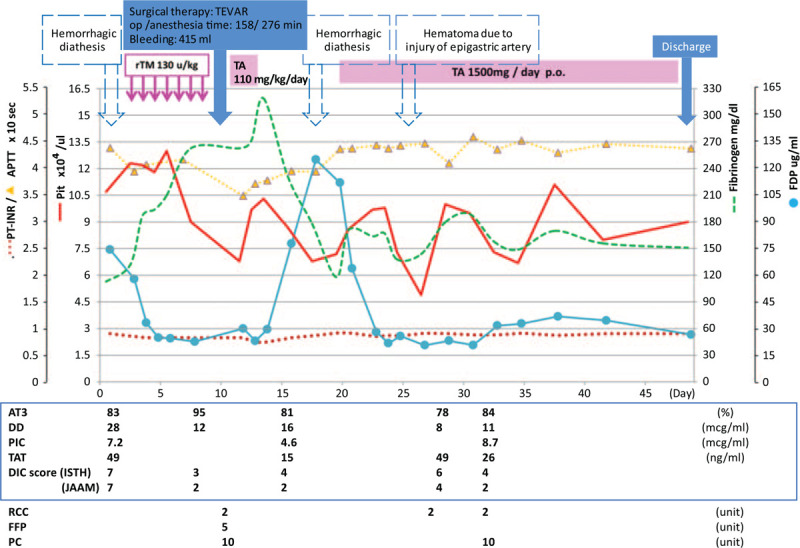
Perioperative course: clinical findings, coagulation function test trends, and anti-DIC treatment. APTT = activated partial thromboplastin, AT3 = antithrombin III, DD = D-dimer, FDP = fibrinogen degradation product, FFP = fresh-frozen-plasma, PC = platelet concentrates, PIC = plasmin-α2 plasmin inhibitor complex, Plt = platelet levels, PT-INR = prothrombin time-international normalized ratio, RCC = red cell concentrates, rTM = recombinant thrombomodulin, TA = tranexamic acid, TAT = thrombin–antithrombin complex.

Figure 2 shows the treatment course and changes in laboratory data after admission. Preoperative examination did not detect endogenous factors, such as malignancy, that might contribute to DIC. Therefore, aortic aneurysm was determined to be the cause of consumptive DIC. Surgical resection was planned due to the expected rupture of the rapidly expanding aneurysm, but according to the JapanSCORE (Japanese Adult Cardiovascular Surgery Database^[12]^), the risk of death from the 30-day surgery was estimated to be 33.6%. Therefore, thoracic aortic stent grafting was planned after anti-DIC treatment and reduction in the bleeding symptoms.

Because the use of heparin alone would have increased bleeding in hyperfibrinolytic DIC, we chose a short-term anticoagulation treatment with rhTM (Asahi Kasei Pharma, Tokyo).

The patient's informed consent was obtained following explanation of the administration procedure. In accordance with the dosage and route of administration for DIC, we initially administered rhTM intravenously at a dose of 130 U/kg/kg (standard dose 380 U/kg/d) in a daily single-drop intravenous injection for 7 consecutive days because renal dysfunction was observed. After the treatment was started, the serum FDP level gradually declined. On the seventh day of treatment, the score improved to 2 points. During the treatment, known rare adverse events associated with rhTM therapy, such as intracranial, gastrointestinal, or pulmonary hemorrhage, were not observed.

Ten days after admission, the patient underwent thoracic aorta stent-graft treatment under general anesthesia. While the amount of intraoperative bleeding was 415 mL, a decrease in the platelet count to 68,000/μL was recognized; therefore, replacement therapy using blood products was conducted. In addition, tranexamic acid was administered at 110 mg/kg/h via continuous infusion to prevent excessive fibrinolysis, both intraoperatively and 1 day postoperatively. The inflammatory response peaked on day 2, subsequently improving. Infectious symptoms were not present; however, bleeding from the puncture site of the blood vessel, and hematoma formation due to arterial injury to the hypogastric wall, were exhibited from postoperative days 5 to 9.

On day 15, the platelet count was 45,000/μL, while fibrinogen and FDP levels were 120 and 120 μg/mL, respectively; excessive fibrinolysis associated with prolonged chronic DIC was suspected. Platelet concentrate was transfused, and tranexamic acid was orally administered to the patient; her symptoms gradually improved, and she was ambulatory when discharged on postoperative day 49.

## 3 Discussion

DIC associated with thoracic aortic aneurysms is characterized by a slow and progressive activation of the coagulation fibrinolytic system and a long-lasting pathology.

Its mechanism involves activation of intrinsic coagulation factors in the tissues underlying the vascular endothelial cells, as well as activation of extrinsic coagulation factors. The former is caused by blood flow abnormalities in the injured vascular walls of aneurysmal regions; the latter is caused by contact between blood and tissue factors in the aortic wall, or cytokine release via infiltrating inflammatory cells, which induce intermittent or continuous thrombus formation and excessive fibrinolysis.

PAI-1 is released into the plasma from the vascular endothelial cells, leading to the suppression of fibrinolysis; therefore, these activations are thought to be slow.^[2,13]^

However, at this stage of the disease, if a patient's condition is complicated by bleeding due to an extrinsic cause, excessive tissue-type plasminogen activator is released from the vascular endothelial cells. Consumption of PAI-1 and α2-plasmin inhibitor is increased due to the formation of a large amount of fibrin, fibrinolysis significantly increases, and fibrinolytic activation may cause decompensated/hemorrhagic fibrinolytic DIC.

In our case, the patient had severe hemorrhage and decompensated/hemorrhagic DIC after an invasive test with arterial puncture.

The primary treatment for aortic aneurysm complicated by DIC is surgical resection of the aneurysm (the cause of the DIC)^[14]^; therefore, if possible, surgery should be performed at the compensated/asymptomatic stage.

However, if aneurysm resection is performed under cardiopulmonary bypass at the stage of markedly increased fibrinolysis, as in the present case, or at the stage of compensated and hemorrhagic DIC, life-threatening hemorrhagic complications are likely to occur due to the consumption of platelets and clotting factors, the use of heparin, and the increased systemic inflammatory response associated with blood contact with foreign bodies and cardiopulmonary ischemia-reperfusion.

Therefore, it is advisable to perform surgery after the hemorrhagic disease resolves with alternative therapies such as anticoagulants, antifibrinolytic drugs, and transfusion therapy.

However, in our case, we were forced to control the DIC and perform a less invasive surgery within a short period because the aortic aneurysm dilated rapidly and had a very high risk of rupture.

Heparin has been widely used for the treatment of DIC. However, there are individual differences in its anticoagulant effect, and there have been reports of cases in which heparin administration did not produce any effect in the short term, or in which hemorrhage occurred despite improvement in DIC on blood tests.^[15]^

Nafamostat mesilate and gabexate mesilate, synthetic thrombin agents, are proteolytic enzyme inhibitors, which inhibit not only coagulation activity but also fibrinolytic activity, and are effective treatments for hyperfibrinolytic DIC caused by aortic aneurysms. However, in addition to the need for continuous infusion, there have been cases of side effects of hyperkalemia that have forced the discontinuation of treatment.^[16]^

Thus, we explored therapeutic strategies, such as using rhTM to control DIC preoperatively and opting for a surgical technique that avoids cardiopulmonary bypass.

In addition to binding to thrombin and exerting an antithrombin effect, rhTM is a coagulation inhibitor that inhibits thrombin production by dramatically activating protein C and inactivating factors Va and VIIIa with protein S as a cofactor. Given that DIC withdrawal rates and the risk of bleeding during treatment were reported to be superior to heparin for DIC caused by blood cancer and infection,^[17]^ it was anticipated that it would also be effective for DIC derived from aortic aneurysms that present with the same hyperfibrinolytic form as blood cancer.

In our case, during the 7-day administration period, the DIC score improved from 7 to 3 points; hemorrhagic complications were not observed. Prolonged DIC may further aggravate a patient's systemic condition and result in treatment-resistant DIC; therefore, if controlling the DIC using rhTM over a short-term period is possible in patients with aneurysm or aortic dissection complicated by decompensated/hemorrhagic DIC, therapeutic strategies including surgical treatment are suggested.^[18,19]^

Aortic stent-graft treatment is widely used as a treatment for thoracic aortic aneurysm and aortic dissection, mainly in Europe and the United States, due to its minimal invasiveness and therapeutic outcomes; this treatment was introduced to Japan in 2008. Postoperative occurrence of consumptive coagulopathy is reportedly low due to thrombosis of the aneurysm and blocked blood flow^[20,21]^; however, it is possible for the coagulation and fibrinolytic systems to be activated at the contact points between the stent graft and blood flow. In preoperative cases of coagulopathy (as presented in this report) or aortic dissection with a large false lumen, remarkable hyperfibrinolysis has been reported to occur postoperatively.^[22,23]^

Although antifibrinolytic treatment was performed at an early stage in this patient, recurrence of DIC was recognized. Even after aortic stent-graft treatment, it is desirable to achieve strict control of the coagulation and fibrinolytic systems.

There were still no randomized, controlled trials of drug therapy for hyperfibrinolytic DIC associated with aortic aneurysms or aortic dissections, and currently no clear guidelines on when to move from drug therapy to surgical treatment.

Because this was only a single-case experience, the response to treatment with rhTM relative to other conventional drugs remains unclear. In the future, new therapies targeting protein C activation using rhTM may play an important role in the treatment of hyperfibrinolytic DIC associated with aortic aneurysms or aortic dissection.

In cases of thoracic aortic aneurysm accompanying chronic DIC, decompensated/hemorrhagic DIC can develop, resulting in severe bleeding diathesis.

Therefore, as shown in our case, preoperative therapy of rhTM for patients who develop thoracic aortic aneurysm with hyperfibrinolytic DIC can reduce the DIC within a short period without the occurrence of hemorrhagic complications, thus providing survival benefits.

## Acknowledgments

The authors thank the nursing staff of the Intensive Care Unit in Saga University Hospital for their assistance. The authors thank Editage (www.editage.com) for English language editing.

## Author contributions

**Conceptualization:** Yoshinori Tanigawa, Yasutaka Yamada, Kimihide Nakamura, Tomoko Yamashita, Akira Nakagawachi.

**Data curation:** Yoshinori Tanigawa, Yasutaka Yamada, Kimihide Nakamura, Tomoko Yamashita, Akira Nakagawachi.

**Formal analysis:** Yoshinori Tanigawa, Tomoko Yamashita, Akira Nakagawachi.

**Funding acquisition:** Yoshinori Tanigawa.

**Investigation:** Yoshinori Tanigawa, Yoshiro Sakaguchi.

**Methodology:** Yoshinori Tanigawa, Tomoko Yamashita, Akira Nakagawachi.

**Project administration:** Yoshinori Tanigawa, Tomoko Yamashita, Akira Nakagawachi.

**Resources:** Yoshinori Tanigawa, Yoshiro Sakaguchi.

**Software:** Yoshinori Tanigawa, Tomoko Yamashita, Akira Nakagawachi.

**Validation:** Yoshinori Tanigawa.

**Visualization:** Yoshinori Tanigawa.

**Writing – original draft:** Yoshinori Tanigawa.

**Writing – review & editing:** Yoshinori Tanigawa, Tomoko Yamashita, Akira Nakagawachi.

## References

[1] Fine NL Applebaum J Elguezabal A. Multiple coagulation defects in association with dissecting aneurysm. Arch Intern Med 1967;119:522–6. 6024665

[2] Fisher DF Jr Yawn DH Crawford ES. Preoperative disseminated intravascular coagulation associated with aortic aneurysms. A prospective study of 76 cases. Arch Surg 1983;118:1252–5. 6639333 10.1001/archsurg.1983.01390110010002

[3] Asakura H Takahashi H Tsuji H. Post-marketing surveillance of thrombomodulin alfa, a novel treatment of disseminated intravascular coagulation—safety and efficacy in 1,032 patients with hematologic malignancy. Thromb Res 2014;133:364–70. 24440141 10.1016/j.thromres.2013.12.033

[4] Matsushita T Watanabe J Honda G. Thrombomodulin alfa treatment in patients with acute promyelocytic leukemia and disseminated intravascular coagulation: a retrospective analysis of an open-label, multicenter, post-marketing surveillance study cohort. Thromb Res 2014;133:772–81. 24636871 10.1016/j.thromres.2014.02.025

[5] Ikezoe T Takeuchi A Isaka M. Recombinant human soluble thrombomodulin safely and effectively rescues acute promyelocytic leukemia patients from disseminated intravascular coagulation. Leuk Res 2012;36:1398–402. 22917769 10.1016/j.leukres.2012.08.012

[6] Levi M Vincent JL Tanaka K. Effect of a recombinant human soluble thrombomodulin on baseline coagulation biomarker levels and mortality outcome in patients with sepsis-associated coagulopathy. Crit Care Med 2020;48:1140–7. 32697484 10.1097/CCM.0000000000004426 PMC7365672

[7] Saito H Maruyama I Shimazaki S. Efficacy and safety of recombinant human soluble thrombomodulin (ART-123) in disseminated intravascular coagulation: results of a phase III, randomized, double-blind clinical trial. J Thromb Haemost 2007;5:31–41. 17059423 10.1111/j.1538-7836.2006.02267.x

[8] Yamakawa K Aihara M Ogura H. Recombinant human soluble thrombomodulin in severe sepsis: a systematic review and meta-analysis. J Thromb Haemost 2015;13:508–19. 25581687 10.1111/jth.12841

[9] Ikezoe T Yang J Nishioka C. Thrombomodulin protects endothelial cells from a calcineurin inhibitor-induced cytotoxicity by upregulation of extracellular signal-regulated kinase/myeloid leukemia cell-1 signaling. Arterioscler Thromb Vasc Biol 2012;32:2259–70. 22796578 10.1161/ATVBAHA.112.251157

[10] Vincent JL Ramesh MK Ernest D. A randomized double-blind, placebo-controlled, Phase 2b study to evaluate the safety and efficacy of recombinant human soluble thrombomodulin, ART-123, in patients with sepsis and suspected disseminated intravascular coagulation. Crit Care Med 2013;41:2069–79. 23979365 10.1097/CCM.0b013e31828e9b03

[11] Gando S Iba T Eguchi Y. A multicenter, prospective validation of disseminated intravascular coagulation diagnostic criteria for critically ill patients: comparing current criteria. Crit Care Med 2006;34:625–31. 16521260 10.1097/01.ccm.0000202209.42491.38

[12] Motomura N Miyata H Tsukihara H. Japan Cardiovascular Surgery Database Organization. Risk model of thoracic aortic surgery in 4707 cases from a nationwide single-race population through a web-based data entry system: the first report of 30-day and 30-day operative outcome risk models for thoracic aortic surgery. Circulation 2008;118:S153–159. 18824747 10.1161/CIRCULATIONAHA.107.756684

[13] Miyahara S Yasu T Yamada Y. Subcutaneous injection of heparin calcium controls chronic disseminated intravascular coagulation associated with inoperable dissecting aortic aneurysm in an outpatient clinic. Intern Med 2007;46:727–32. 17541224 10.2169/internalmedicine.46.6155

[14] Aboulafia DM Aboulafia ED. Aortic aneurysm-induced disseminated intravascular coagulation. Ann Vasc Surg 1996;10:396–405. 8879398 10.1007/BF02286787

[15] Oba J Shiiya N Matsui Y. Preoperative disseminated intravascular coagulation (DIC) associated with aortic aneurysm—does it need to be corrected before surgery? Surg Today 1995;25:1011–4. 8645932 10.1007/BF00311684

[16] Kawano H Hata T Uda A. Use of rivaroxaban for the effective management of disseminated intravascular coagulation associated with abdominal aortic aneurysm. Intern Med 2015;54:2625–8. 26466700 10.2169/internalmedicine.54.4942

[17] Saito H Maruyama I Shimazaki S. Efficacy and safety of recombinant human soluble thrombomodulin (ART-123) in disseminated intravascular coagulation: results of a phase III, randomized, double-blind clinical trial. J Thromb Haemost 2007;5:31–4. 17059423 10.1111/j.1538-7836.2006.02267.x

[18] Iyama S Sato T Murase K. Intermittent administration of recombinant human soluble thrombomodulin successfully controlled chronic disseminated intravascular coagulation in a patient with dissecting aortic aneurysm on an outpatient basis. Blood Coagul Fibrinolysis 2012;23:548–50. 22732250 10.1097/MBC.0b013e32835510d6

[19] Hoshina K Shigematsu K Hosaka A. The effect of recombinant human soluble thrombomodulin on disseminated intravascular coagulation in an abdominal aortic aneurysm. Blood Coagul Fibrinolysis 2014;25:389–91. 24378974 10.1097/MBC.0000000000000031

[20] Rowlands TE Norfolk D Homer-Vanniasinkam S. Chronic disseminated intravascular coagulopathy cured by abdominal aortic aneurysm repair. Cardiovasc Surg 2000;8:292–4. 10840209 10.1177/096721090000800411

[21] Monaco M Di Tommaso L Stassano P. Impact of blood coagulation and fibrinolytic system changes on early and mid term clinical outcome in patients undergoing stent endografting surgery. Interact Cardiovasc Thorac Surg 2006;5:724–8. 17670694 10.1510/icvts.2006.136507

[22] Cross KS Bouchier-Hayes D Leahy AL. Consumptive coagulopathy following endovascular stent repair of abdominal aortic aneurysm. Eur J Vasc Endovasc Surg 2000;19:94–5. 10706846 10.1053/ejvs.1999.0970

[23] Shimazaki T Ishimaru S Kawaguchi S. Blood coagulation and fibrinolytic response after endovascular stent grafting of thoracic aorta. J Vasc Surg 2003;37:1213–8. 12764267 10.1016/s0741-5214(02)75323-8

